# Correlation between CTMP expression levels and resistance to trastuzumab in HER2 + metastatic breast cancer

**DOI:** 10.1007/s12672-025-03210-x

**Published:** 2025-07-16

**Authors:** Mania Makhoul, Maher Saifo, Fariz Ahmad, Jumana Saleh

**Affiliations:** 1 Department of Biochemistry and Microbiology, Faculty of pharmacy, Damascus University, Damascus, Syrian Arab Republic; 2 Prof. Medical Oncology, Damascus University, Damascus, Syrian Arab Republic; 3 Head of department of pathology, Faculty of medicine, Damascus University, Damascus, Syrian Arab Republic; 4 Dr. Rer. Med. Immunology, Faculty of pharmacy, Damascus University, Damascus, Syrian Arab Republic

**Keywords:** CTMP, Metastatic breast cancer, Trastuzumab resistance

## Abstract

**Background:**

The combination of trastuzumab and chemotherapeutic drugs improves the prognosis of patients with metastatic disease and reduces the mortality. However, trastuzumab resistance has limited the remarkable improvement of this drug. The carboxyl-terminal modulator protein (CTMP) is involved in the regulation of various cancers through positive or negative regulation of Akt. In the HER2-positive SkBR3 breast cancer cell line, CTMP overexpression increases Akt phosphorylation at Thr308 and Ser473. Therefore, CTMP might mediate trastuzumab resistance. The main objective of the paper is to explore the role of CTMP in trastuzumab efficacy in HER2 + metastatic breast cancer (MBC) patients.

**Patients & methods:**

Ninety-six patients received trastuzumab in combination with chemotherapy or hormonal therapy until disease progression. The overall responses of all the patients were assessed as follows: complete response (*n* = 5), partial response (*n* = 36), stable disease (*n* = 24), and progressive disease (*n* = 31).

**Results:**

Immunohistochemistry (IHC) staining was carried out to identify CTMP expression in formalin-fixed paraffin-embedded (FFPE) archival tissue blocks. 58 cases had high CTMP expression levels and 38 cases had low CTMP expression levels. The Mann-Whitney U test showed that CTMP expression was markedly higher in trastuzumab non-responders than in trastuzumab responders (*P* = 0.039). In addition, high CTMP expression was a strong and independent predictor of shorter recurrence-free survival in patients with metastatic breast cancer, as determined by the Kaplan-Meier method.

**Conclusions:**

Based on the results, further examination of CTMP in HER2-enriched (MBC) tissue samples could be helpful in predicting patients at risk of tumor progression and trastuzumab resistance.

## Introduction

Globally, breast cancer (BC) is the most common type of cancer in women (approximately 30%) and serves as the anterior source of mortality [1]. Breast cancer can be classified into three subclasses based on the expression of the estrogen receptor (ER), progesterone receptor (PR), and ERBB2 gene amplification (HER2). Cases that have (ER) and- /or (PR) but not HER2, are classified as HR+/HER2- breast cancer, which accounts for 75% of all subtypes. Approximately 20–25% of cases that express HER2 with or without (HRs) expression are referred to as HER2 + breast cancer. The residual 10–17% of breast cancers is triple-negative breast cancer (TNBC), which does not express any of the ER, PR, or HER2 proteins. These three subtypes have multiple risk profiles and treatment approaches [2].

Human epidermal growth factor receptor 2 (HER2) is a member of the epidermal growth factor receptor family. In metastatic breast cancer (MBC), reports have shown that overexpression of HER2 is related to diminished disease-free survival [3]. Overexpression of HER2 been detected in 20–25% of breast cancer cases [4]. The identification of HER2 status has become an essential stage in breast cancer diagnosis, which is important for prognosis and choice of therapy [5].

Systemic chemotherapy is the standard treatment for MBC. However, chemotherapy plus HER2 combined with targeted therapy has been shown to reduce the high toxicity of chemotherapy. Trastuzumab, the mainstay of success in breast cancer-targeted therapy, is a recombinant humanized monoclonal antibody (mAb) designed to target (HER2) at an extracellular domain. The combination of trastuzumab with chemotherapy drugs enhances the prognosis of cancer and reduces both mortality and cancer frequency [6–8].

The most intensively studied antitumor mechanisms for trastuzumab against cancer cells are: (1) Altering the cellular physiology by the suppression of proliferation [9]; (2) Limitation the formation of aggressive tumor microenvironments by blocking the release of vascular endothelial growth factor (VEGF) [10]; (3) Inhibition HER2 ectodomain cleavage, which reduces production of oncogenic p95HER2 [11]; (4) Activation of innate and adaptive immune responses, via antibody-dependent cellular cytotoxicity (ADCC) [12]; (5) Inhibition DNA synthesis after radiation and impact on key DNA repair system [13]; (6)Trastuzumab enhances the activity of tyrosine kinase-ubiquitin ligase c-Cbl leading to the degradation of HER2 [14]; (7) Trastuzumab blocks the interactions of Src kinase with HER2 causing obstacle HER2 signaling [15].

Despite the clinical efficacy of trastuzumab, a considerable proportion of patients with HER2 + breast cancer experience relapse owing to primary or secondary resistance to trastuzumab. Most patients who initially respond to trastuzumab– based therapy develop resistance within one year of treatment initiation [16]. Several mechanisms involved in trastuzumab resistance have been discovered including: (1) Expression of HER2 carboxyl-terminal fragment (p95HER2). P95HER2 lacks trastuzumab-binding epitopes that disrupt the ability of HER2 to bind trastuzumab [17]. In (MBC) patients treated with trastuzumab, overexpression of p95HER2 was correlated with shorter progression-free survival (PFS) and overall survival (OS) [18]. (2) In BC cells, Mucin-4 hides trastuzumab-binding sites. MUC4 also impedes the interactions between HER2 and its binding partners, and this in turn activates MAPK and PI3K signaling pathways [19]. There are remarkable correlation between enhanced levels of MUC4 and a poor response to trastuzumab in HER2 breast cancer patients [20]. (3) Resistance to trastuzumab may also be related to the activation of HER2 downstream signaling pathways, particularly PI3K/AKT. PI3KCA mutation and PTEN deficiency were detected in approximately 25% of patients with HER2 overexpression [21]. (4) Immune regulatory processes have been proposed as another mechanism of trastuzumab resistance. Programmed death-ligand 1 (PD-L1) suppresses immune effectors by ligating PD-1 [22].

Trastuzumab resistance clinical problem still limits the remarkable improvement of this drug. Despite comprehensive research on the molecular mechanisms responsible for trastuzumab resistance, data remain insufficient to compare the results and draw reliable images to understand this dynamic cellular process.

The carboxyl-terminal modulator protein (CTMP), also known as Them4, is a member of the thioesterase superfamily, member 4 (gene ID 117145 in Entrez/NCBI). In breast cancer studies, CTMP was found to interact with Akt and positively regulate Akt phosphorylation, ultimately promoting cell proliferation [23, 24]. Therefore, CTMP might mediate trastuzumab resistance.

The present study aimed to determine the functional role of CTMP and its association with trastuzumab efficacy in Syrian patients with HER2 + metastatic breast cancer. This article was previously presented as a meeting abstract at the 2024 ASCO Annual Meeting on May 29, 2024.

## Patients and methods

### Study design and participants

Our study was designed as a clinical retrospective observational study of Syrian breast cancer patients attending Al Bairouni University Hospital, between the period 2021–2023. We aimed to evaluate the role of CTMP in trastuzumab resistance in Syrian patients with (HER2 + MBC).

#### Eligibility

The major eligibility criteria were as follows: female age 18 years or older; histologically confirmed metastatic breast cancer; presence of an HER2-positive tumor, defined as immunohistochemistry (IHC) test + 3 or fluorescence in situ hybridization (FISH) test-positive (HER2/CEN-17 > 2.2); at least one measurable lesion, Eastern Cooperative Oncology Group (ECOG) performance status of 0 to 1, depending on the (RECIST 1.1) for response evaluation in solid tumors; the left ventricular ejection fraction (LVEF) was equal to or greater than the lower limit of the normal value; the basic index for blood tests, liver, and kidney function were within the normal range; a life expectancy of at least 12 weeks; and women with central nervous system (CNS) metastases were eligible if they were clinically constant for at least 3 months after cessation of corticosteroid and anticonvulsant therapy.

#### Ineligibility

The following Ineligibility criteria were considered: women who were pregnant or breast-feeding, had previously received systemic therapy for metastatic disease, had gastrointestinal functional disease that could alter the absorption of orally administered vinorelbine, had cardiac dysfunction, and had other types of malignant tumors. Patients for whom detailed clinical information or pathological tissue samples were unavailable were also excluded.

### Treatment program

All the patients received trastuzumab therapy. In addition to trastuzumab, different types of chemotherapeutic agents, such as taxane (docetaxel) or vinorelbine, were administered to patients in standardized doses. Tamoxifen and aromatase inhibitors (letrozole) were administered as hormonal therapy. The patients received a loading dose of 8 mg/kg trastuzumab, followed by a maintenance dose of 6 mg/kg every 21 days. Docetaxel was administered every 21 days at a starting dose of 75 mg/m^2^. The vinorelbine dose was 60 mg/m^2^ D1, D8, 21-day cycles. All drugs were administered intravenously except vinorelbine, which was administered orally. Participants continued to receive trastuzumab plus endocrine therapy or chemotherapy until disease progression, symptomatic deterioration, progression of unmanageable toxicity, cancellation of consent, or death.

### Efficacy assessment

Before entering the study, a baseline assessment was applied to all patients, such as a complete medical history, bone scan, chest CT, abdominal CT, or MRI scan. In cases of partial or complete response, a confirmative CT scan was performed four weeks later, followed by a CT scan every two treatment cycles. All patients were assessed for clinical symptoms at the baseline and before each cycle.

Therapeutic efficacy was assessed consistently every three cycles starting from the first cycle of treatment, according to the RECIST 1.1. Response was defined according to the efficacy criteria: complete response (CR) (disappearance of all tumors for four weeks and over according to radiology), partial response (PR) (≥ 30% reduction in size of tumor lesions for at least four weeks); stable disease (SD) was defined as no change in the size of lesions ≥ 20% and no appearance of new lesions, while progressive disease (PD) was defined as an increase in the size of the existing lesions ≥ 20% or the appearance of any new lesion. The Objective Response Rate (ORR) was defined as the proportion of patients achieving CR or PR, whereas patients with CR, PR, or SD were included in the Clinical Benefit Rate (CBR) for at least three months.

### Data collection

A retrospective survey of the medical records, depending on the study protocol, was conducted. The chart abstraction design was rehashed at monthly patient follow-up. Age, tumor subtype histology, tumor grade, Estrogen Receptor (ER) status, Progesterone Receptor (PR) status, HER2 Receptor status (HER2), Performance status (PS), number of metastatic sites, metastatic location, date of diagnosis, date of relapse, and date of first progression were estimated at the time of MBC diagnosis using a chart abstraction design.

### (HR) detection

Tumors were defined as HR-positive (HR+) if estrogen receptor (ER) or progesterone receptor (PR) expression was ≥ 10% (immunohistochemistry), as per European guidelines.

### HER2 detection

HER2 status was detected using immunohistochemistry (IHC) and/or in situ hybridization (ISH). IHC describes overexpression on a scale of 0–3+. HER2 expression was considered positive if the staining intensity was grade 3 + on IHC or grade 2 + on fluorescence gene amplification. All cancers with an IHC score of 0–1 + or 2 + and a negative FISH/CISH test result were considered as HER2 negative (HER2-). Cancers with an IHC score of 2 + and those without FISH/CISH test results were considered to be HER2 equivocal.

### Immunohistochemical (IHC) analysis for CTMP

Patient-derived formalin-fixed paraffin-embedded (FFPE) archival tissue blocks before starting trastuzumab-based therapy were evaluated by the pathology department of AL Bairouni Hospital to analyze the expression levels of CTMP/THEM4 via IHC. In this method, 4 μm- thick sections were transferred from FFPE tissues to positively charged microscope slides and deparaffinized overnight at 37 ° C after which the sections were soaked for 5 min in three separate xylene solutions. The sections were soaked in two separate 96% ethanol solutions and endogenous tissue peroxidase activity was repressed by 3% H2O2 solution. Antigen retrieval was performed to reveal masked antigens by incubating the slides in 10 mM citric buffer (pH 6.0) and microwaved for 15 min in a glass beaker. After blocking, 1:50 diluted primary anti-CTMP antibodies were dripped onto separate cross sections and incubated for 30 min at room temperature. Followed by applying Biotin polymer-HRP and 3, 3′diaminobenzidine (DAB) peroxidase substrate. Converse staining was performed using Mayer´s hematoxylin. Each test group was run using both positive and negative controls.

### Evaluation of IHC staining

The extent and intensity of the IHC staining were estimated by a board-certified pathologist. The results were categorized on the basis of two parameters: staining intensity was assessed in the range of 0–3 (nil, no staining; 1, weak; 2, moderate; and 3, strong). The extent of staining was assessed as follows (0, no staining; 1, < 35% positive cells; 2, 35–75%; and 3, >75%). By multiplying the staining intensity by the extent, we obtained the IHC staining grade (range: 0–9). Grade 0 was defined as no staining, grades 1 and 2 as weak staining (+ 1), grades 3 and 4 as moderate staining (+ 2), and grades 6 and 9 as strong staining (+ 3). Finally, specimens with no staining and (+ 1) staining were defined as the low CTMP group and those with (+ 2) and (+ 3) staining were defined as the high CTMP group.

### Statistical analyses

Statistical analyses were performed using SPSS version 20.0. Statistical comparisons of the results were performed using Student’s t-test and Mann-Whitney test. Progression-free survival (PFS) rates were calculated using the Kaplan-Meier method, and differences in survival curves were analyzed using log-rank tests.

All tests were two-sided. The significance level was set at *P* < 0.05.

## Results

From September 2021 through February 2023, a total of 125 patients were enrolled at Al Bairouni University Hospital, 96 patients were recruited for this study based on the above inclusion and exclusion criteria. All patients in the present study were females with HER2-positive metastatic breast cancer (stage IV), had at least one measurable lesion according to the Response Evaluation Criteria in Solid Tumors (RECIST). Of the 29 patients who were excluded from participating in the study, 24 either did not meet the criteria for inclusion or met the criteria for exclusion, 2 withdrew consent, 2 were not evaluated due to drug shortage or disruption, and 1 was lost to follow-up.

The demographic characteristics of (HER2 + MBC) patients included in the current study are outlined in Table 1. The average age at diagnosis was 48 years, and there was an almost even distribution of cases according to age. Most patients had invasive ductal carcinoma (97.9%). The efficacy assessments of the patients are summarized in Table 2.

Comparing the groups, there were no significant differences in laterality, histologic type, hormone receptors status, metastatic sites, and number of metastatic sites. A statistically significant association was identified between CTMP overexpression and tumor grade (*P* = 0.047) and the highest frequency of CTMP overexpression was observed in cases with Grade III (67.7%) followed by Grade II category (47.1%).

**Table 1:** Demographic characteristics of 96 (HER2 + MBC) patients registered in the study

Feature	All patients *N* (%)
Age at diagnosis	
Age ≤ 48	50 (52.1)
Age > 48	46 (47.9)
Tumor site	
Right	46 (47.9)
Left	49 (51.0)
Right & Left	1 (1.0)
Tumor subtype histology	
IDC	94 (97.9)
ILC	2 (2.1)
Tumor Grade	
GII	34 (35.4)
GIII	62 (64.6)
Hormone receptor status	
ER-/ PR-	43 (44.8)
ER+/ PR-	14 (14.6)
ER+/ PR+	38 (39.6)
ER-/ PR+	1 (1.0)
Number of metastatic sites	
1	48 (50)
2	43 (44.8)
3	4 (4.2)
4	1 (1.0)
Metastatic sites	
Bone	48 (32.0)
Liver	32 (21.3)
Lung	39 (26.0)
Skin	3 (2.0)
Brain	4 (2.7)
Pleural effusion	6 (4.0)
Lymph node	18 (12.0)

*MBC* metastatic breast cancer; *IDC* invasive ductal carcinoma; *ILC* invasive lobular carcinoma; *ER* estrogen receptor; *PR* progesterone receptor

**Table 2:** Overall response of 96 (HER2 + MBC) patients after Trastuzumab-based therapy

Response to treatment	
CBR no. (%)	65 (67.7)
ORR no. (%)	41 (42.7)
CR no. (%)	5 (5.2)
PR no. (%)	36 (37.5)
SD no. (%)	24 (25.0)
PD no. (%)	31 (32.3)

*CBR* clinical benefit rate; *ORR* objective response rate; *CR* complete response; *PR* partial response; *PD* progressive disease

### CTMP expression in metastatic breast cancer patient tissues

**Fig. 1:**
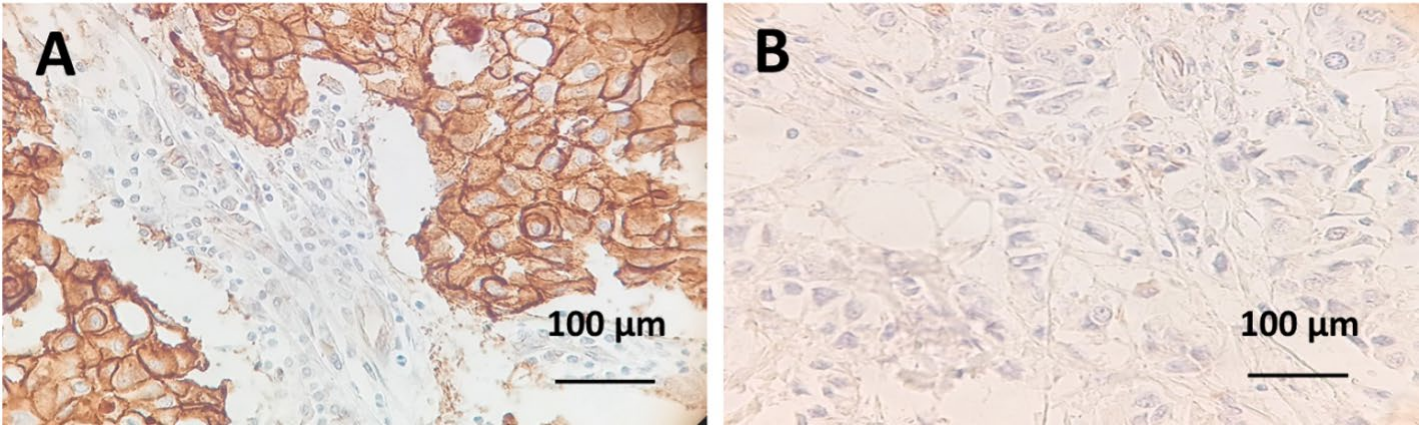
Immunohistochemical staining of CTMP in breast cancer specimens. **A** High immunostaining for CTMP. **B** Low immunostaining for CTMP

To determine the clinical relevance of CTMP expression in MBC tissues, IHC staining for CTMP was performed on 96 paraffin-embedded tissue samples harvested from MBC patients. Representative IHC images are shown in Fig. 1. CTMP staining revealed variable expression levels. CTMP IHC staining was scored on a scale of 0 to + 3 and subdivided into low (0 and + 1) and high (+ 2 and + 3) expression groups. 38 Cases (39.6%) had low CTMP staining and 58 (60.4%) had high CTMP staining.

### Patients with higher CTMP expression tended to be resistant to trastuzumab-based therapy

**Fig. 2:**
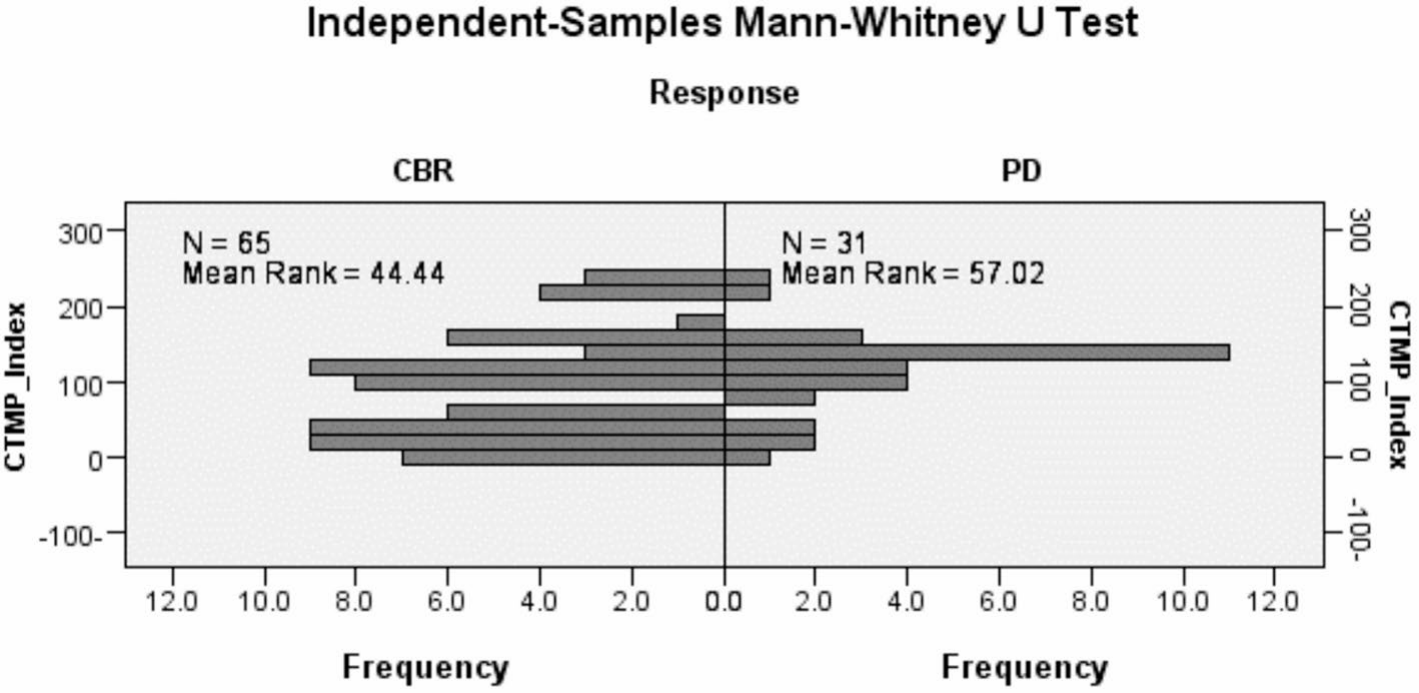
The expression levels were compared between CBR and PD by Mann-Whitney U Test. In each sample, 100 cells were scored from 0 to 3 and the sum was taken as the staining index

**Fig. 3:**
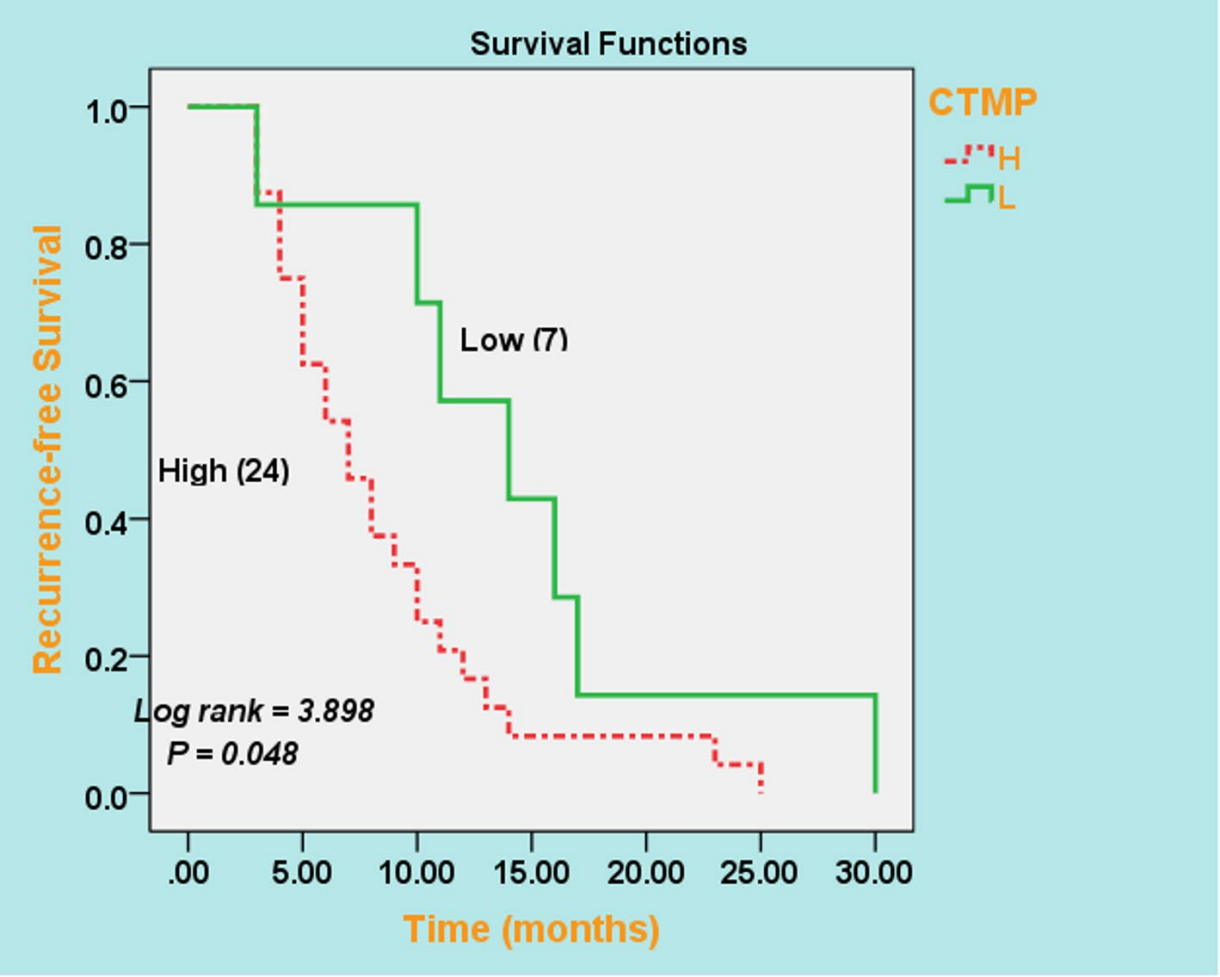
Kaplan-Meier plots for recurrence-free survival based on CTMP expression levels as determined by IHC in tumor tissue samples

To determine the role of CTMP in trastuzumab resistance, we examined CTMP in samples from 96 patients who received trastuzumab as adjuvant treatment. Among these, 65 patients had a clinical benefit rate (CBR) and 31 patients experienced progressive disease (PD). The CTMP staining index was compared between (CBR group) and (PD groups) by Mann-Whitney U test. The data showed that CTMP staining index was significantly higher in (PD group) than in (CBR group) (*P* = 0.039) (Fig. 2). Furthermore, stratified survival analysis revealed that MBC patients with high CTMP expression had shorter recurrence-free survival than those with low CTMP expression, as evidenced by Kaplan-Meier curves (*P* = 0.048) (Fig. 3). The relative risk of trastuzumab resistance at the highest versus the lowest CTMP expression level was 2.2%.

## Discussion

The serine/threonine kinase AKT, also known as protein kinase B, plays a pivotal role in the PI3K/AKT signaling pathway. AKT is involved in the regulation process of many hallmarks of malignancy, including growth and invasiveness of tumor cells [25]. AKT is a member of the ACG family and comprises an N-terminal PH domain, linker region, catalytic domain, and a C-terminal regulatory domain [26]. PI3K is activated by many hubs including receptor tyrosine kinases (RTKs), RAS-related GTPases, and heterotrimeric G proteins [27]. Activated (PI3Ks) enhances the phosphorylation of Phosphatidyl-inositol-4, 5-bisphosphate (PIP2) to form the second messenger Phosphatidyl-inositol-3, 4, 5-triphosphate (PIP3). As PIP3 binds to AKT, Akt undergoes binary phosphorylation by PDK1 and mTOR complex2 [28]. Aberrant activation of the PI3K/AKT/mTOR pathway acts as a tumor trigger in HER2 + BC due to mutations in PI3KCA or PTEN. Consequently, this overactivation results in primary and acquired resistance to HER2-targetad therapies [15].

27-kDA CTMP has been characterized in a yeast two-hybrid screen as an Akt-interacting protein [29]. Some studies have shown that CTMP binds to AKT and negatively regulates its activity in many diseases, including lung cancer, colorectal cancer, pancreatic cancer, liver cancer, diabetes, cardiovascular disease, myogenic differentiation, and amyotrophic lateral sclerosis. Conversely, CTMP can also act as a positive regulator of Akt in breast cancer and nasopharyngeal carcinoma [30].

In this study, we aimed to explore the effect of CTMP on trastuzumab resistance in Syrian patients with (HER2 + MBC). The Mann-Whitney U test revealed, that CTMP expression was obviously higher in trastuzumab non-responders (PD group) than in trastuzumab responders (CBR group) (*P* = 0.039). In addition, high CTMP expression was a strong and independent predictor of shorter recurrence-free survival in patients with metastatic breast cancer, as determined by the Kaplan-Meier method. Collectively, examining CTMP in HER2-enriched (MBC) tissues could be helpful in predicting patients at risk of tumor progression and trastuzumab resistance.

## Conclusion

In conclusion, HER2 + metastatic breast cancer patients with high CTMP expression levels tend to be resistant to trastuzumab-based therapy. Further detailed investigations may raise the possibility of the clinical use of the CTMP protein as a candidate biomarker. Therefore, targeting CTMP signaling may pave the way for tailoring novel therapeutic approaches that can achieve better blockade of HER2 family receptor signaling to overcome trastuzumab resistance for effective control of metastatic breast cancer.

## Data Availability

All data supporting the findings of this study are available within the paper.
